# Supplementary material for: QuATON: Quantization Aware Training of Optical Neurons
Source: Res Sq. 2024 Mar 27:rs.3.rs-4076842. Preprint. [Version 1] doi: 10.21203/rs.3.rs-4076842/v1 (PMC10996779; doi:10.21203/rs.3.rs-4076842/v1)
Supplement: Supplement 1 [file NIHPPrs4076842v1-supplement-1.pdf]

Progressive Sigmoid Quantization Framework for Optical Neural Architectures

Supplementary Material

A Simulating Light Propagation

This section gives a detailed information on light propagation through the D2NNs and backpropagation to optimize the D2NN and PSQ paratmeters. During the QAT, the forward propagation through the immediate output of the nth layer is given by Eq. (6), which is given below.

$$E^n_{out}[x,y] = F_n\left(E^n_{in}[x,y]\right) = E^n_{in}[x,y]\exp\left(jQ_s\left(\varphi_n[x,y],\tau_n\right)\right) \tag{S1}$$

Here, $E^n_{out}[x,y]$ is the modulated output field from the nth layer of the D2NN, $E^n_{in}[x,y]$ is the input field to the nth layer. $[x,y]$ discrete coordinates correspond to the location of a neuron on the D2NN layer. $\varphi[x,y]$ is the phase coefficient of a neuron, $Q_s(.)$ is the PSQ function, and τ_n is the temperature factor of layer n. For propagating this field to the next layer, we use the Rayleigh-Sommerfeld diffraction formulation [27, ch. 3.5]. Using this, the input field to neuron at $[x_i,y_i]$ of the $(n+1)^{\text{th}}$ layer is given by

$$E^{n+1}_{in}[x_i,y_i] = \sum_{x,y} E^n_{out}[x,y]\Delta A^n[x,y] w^n_{x,y,i,z}, \tag{S2}$$

where $\Delta A^n[x,y]$ is the area of the neuron at $[x,y]$ and

$$w^n_{x,y,i,z} = \left(\frac{z}{r^2}\right)\left(\frac{1}{2\pi r}+\frac{1}{j\lambda}\right)\exp\left(j\frac{2\pi r}{\lambda}\right). \tag{S3}$$

In this, z is the distance between the two layers, λ is the wavelength, and $r = r_{x,y,i,z} = \sqrt{(x_i-x)^2+(y_i-y)^2+z^2}$. For the simplicity, we write the propagation in Eq. (S2) as

$$E^{n+1}_{in}[x_i,y_i] = G\left(E^n_{out}[x,y],x_i,y_i,z\right). \tag{S4}$$

For the special cases $n=0$ (i.e. propagation of input field to the first layer), and $n=N$ (i.e. propagation from the last layer to the detector) Eq. (S4) becomes,

$$\begin{aligned} E^1_{in}[x_i,y_i] &= G_{in}\left(E_{in}[x,y],x_i,y_i,z_{in}\right) \\ E_{out}[x_i,y_i] &= G_{out}\left(E^N_{out}[x,y],x_i,y_i,z_{out}\right), \end{aligned} \tag{S5}$$

Here z_{in} and z_{out} are the distance between the input plane and the first layer, and the distance between the last layer and the detector. In the simulation, we use the angular spectrum method[27,34] for an efficient implementation of this propagation.

For the optimization process, we need to compute the gradients of the field, with respect to the D2NN parameters and k_n parameters of each layer (used to compute the temperature of each layer as described in the Methods and Materials section). These gradients can be computed using the chain rule as following.

$$\begin{aligned} \frac{\partial E^{n+1}_{in}}{\partial \varphi_n} &= jG'\left(E^n_{out},z\right)E^n_{in}e^{jQ_s(\varphi_n,\tau_n)}\partial_{\varphi_n}Q_s\left(\varphi_n,\tau_n\right) \\ \frac{\partial E^{n+1}_{in}}{\partial k_n} &= jG'\left(E^n_{out},z\right)E^n_{in}e^{jQ_s(\varphi_n,\tau_n)}\partial_{\tau_n}Q_s\left(\varphi_n,\tau_n\right)\partial_{k_n}\tau_n \end{aligned} \tag{S6}$$

The partial derivatives terms in the above equations are computed as

$$\begin{aligned} \frac{\partial Q_s}{\partial \varphi_n} &= \Delta\tau_n\sum_{i=0}^{N-2} sig'\left(\tau_n\left(\varphi_n-\beta_i\right)\right) \\ \frac{\partial Q_s}{\partial \tau_n} &= \Delta\sum_{i=0}^{N-2}\left(\varphi_n-\beta_i\right)sig'\left(\tau_n\left(\varphi_n-\beta_i\right)\right) \\ \frac{\partial \tau_n}{\partial k_n} &= \frac{-1}{(|k_n|+\gamma)^2}\frac{d|k_n|}{dk_n}, \end{aligned} \tag{S7}$$

where $\beta_i = l + (i + 0.5)\Delta$ and $sig'(.)$ is the derivative of the sigmoid function given by

$$sig'(x) = sig(x)\,(1 - sig(x))\,. \tag{S8}$$

All the other symbols have the same meanings as in the main text. Note that the partial derivatives with respect to k_n are computed only for the PSQ-LT method. We implement the entire training pipeline using pytorch automatic differentiation[35].

B Training Details

This section gives additional details regarding the training process and specifications of D2NNs considered in the study.

B.1 D2NN Specifications

For both the tasks, D2NNs operating in the wavelength $\lambda = 632.8$ nm with neuron size of $0.5\lambda \times 0.5\lambda$ are used. For the MNIST digit dataset (for both tasks), 7-layer D2NNs are used with 64×64 neurons per layer. Input field of view (FoV) is the same as a layer size. The distance between two adjacent layers and the distance between the input FoV and the first layer are set to 5.3λ, where the distance between the final layer and the detector is set to 9.3λ.

For the tinyimagenet and red blood cell datasets (for all-optical QPI), 5-layer D2NNs with 200×200 neurons per layer are used. The input FoV in these cases are set to 2.5 times smaller than D2NN layers, and the spacing between the layers, between the input FoV and the first layer, and between the last layer and the detector are all set to 40λ. These distances are same for the CIFAR10 dataset (all-optical classification). However, D2NNs with 7 layers, each having 64×64 neurons and same size as the input FoV are used.

B.2 Training and Performance Evaluation

All the models are trained using the Adam optimizer[36]. During the training process, PSQ is used to quantize the D2NN parameters. However, during validation and testing phases, the trained parameters are quantized using the hard-quantization function.

Each dataset considered is seperated into three partitions; train, validation, and test sets. The models are trained on the train set for 100 epochs (200 epochs for CIFAR10) using full-precision parameters. Starting from this model, QAT is performed for another 100 epochs. Then the trained model of the epoch with the highest performance for the validation set is chosen and evaluated on the test set. The same method is used to evaluate the performance for other QAT methods considered.

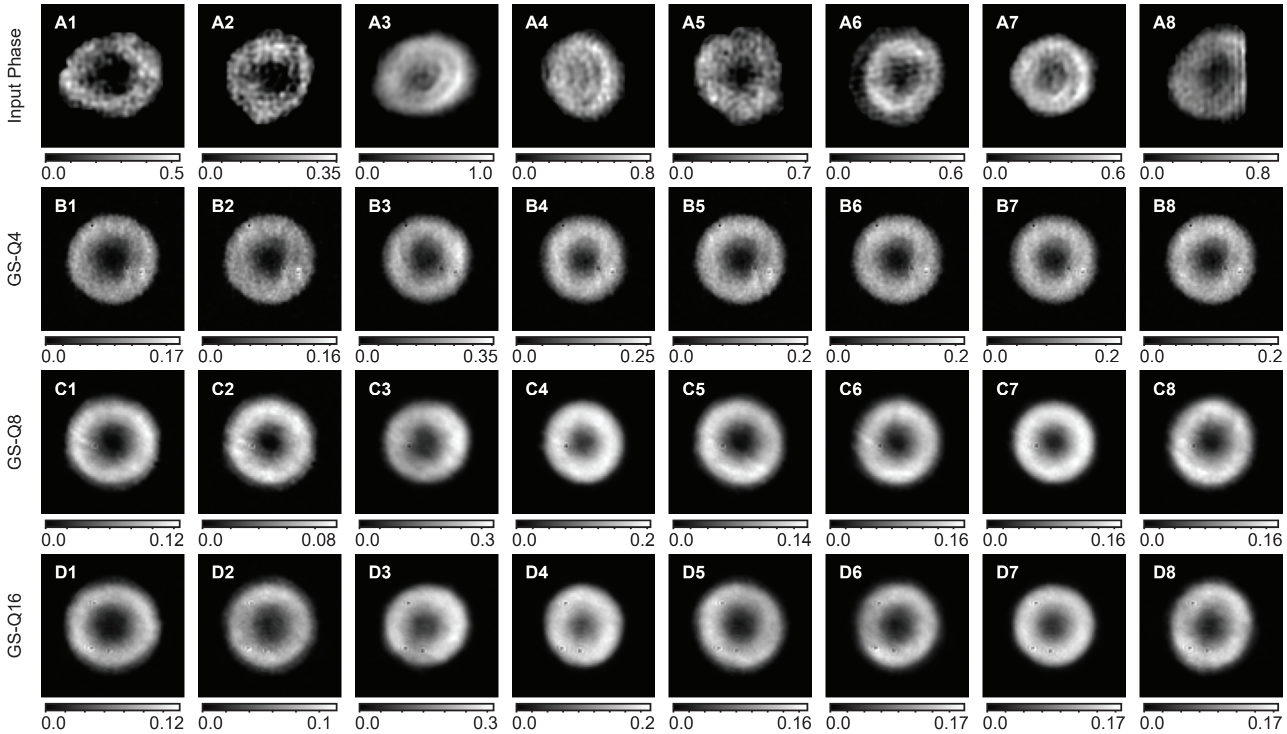

Figure S1. Mode collapse of D2NNs trained using GS for RBC dataset: A1)-A8) show eight randomly selected examples from the RBC test set. Subsequent rows show the resulting output intensities from D2NNs trained using GS with 4-level (**B1-B8**), 8-level (**C1-C8**), and 16-level (**D1-D8**) quantized phase weights. Although the inputs have different morphologies, each D2NN gives similar outputs to all the inputs.

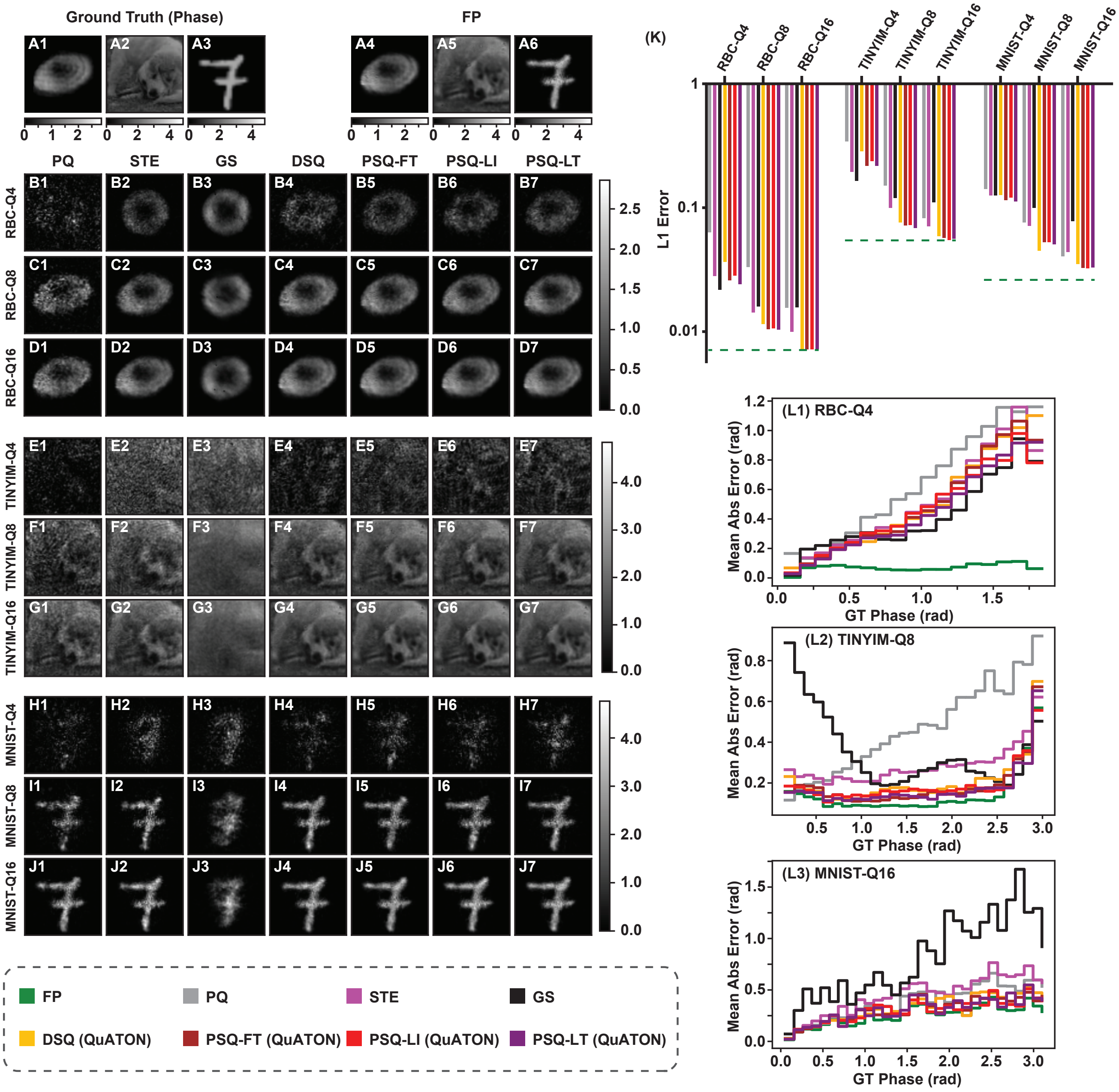

Figure S2. **All-optical quantitative phase imaging results (additional examples): A1)-A3)** show three examples of the phase of the incoming wave to the D2NN for the three datasets considered. **A4-A6)** show the output intensities $\times\pi$ for the D2NNs trained with full precision (FP) weights. Rows **B)-J)** show the QPI results for quantization-aware trained D2NNs for each of the examples. Each row named as $\boldsymbol{x}$**-Q**$\boldsymbol{n}$ shows the results for dataset $\boldsymbol{x} \in \{$RBC, TINYIM, MNIST$\}$, using D2NNs trained with $\boldsymbol{n}$-level quantized weights ($\boldsymbol{n} \in \{4, 8, 16\}$). Each column corresponds to different QAT methods considered which are stated above the row B). Note that all the results are given as output intensity $\times\,\pi$. **K)** shows the comparison of the mean L1 error of the predictions over the test set of each dataset. **L1)-L3)** show mean absolute phase error variation against ground truth phase for **RBC-Q4, TINYIM-Q8,** and **MNIST-Q16** cases respectively. These plots are shown for the given examples in the figure.

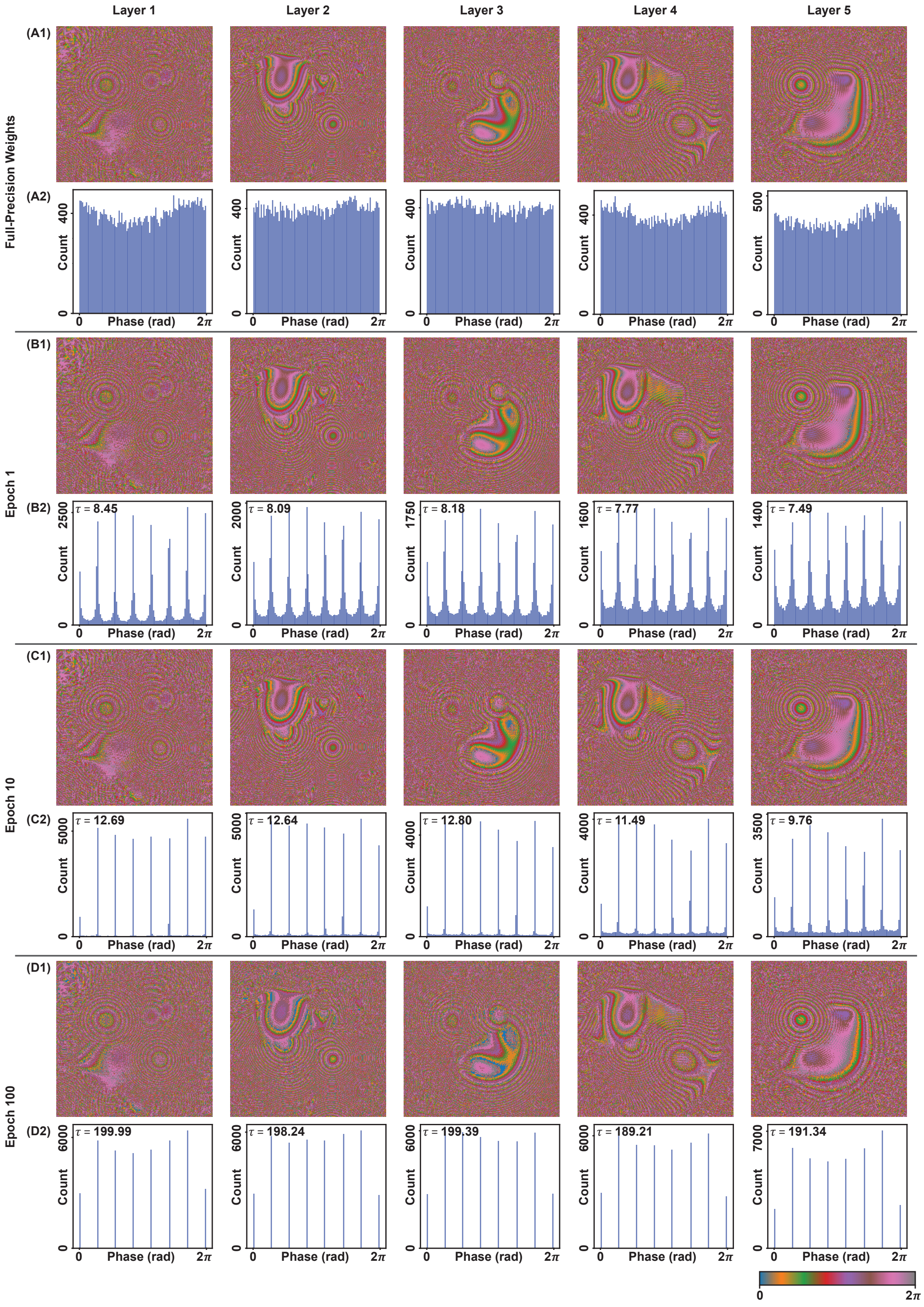

Figure S3. **Progressive training for quantization (For TINYIM-Q8 case using PSQ-LT method): A1)** and **A2)** shows full-precision initialization and the weight distributions of the phase maps for each D2NN layer. Rows **B)** - **D)** shows the phase maps and weight distributions for epochs 1, 10, and 100 respectively during progressive training. In each epoch, the learned temperature factor for each layer is shown in the top-left corner of the histograms.
